# Molecular Basis of Lipid Metabolism in *Oryza sativa* L.

**DOI:** 10.3390/plants13233263

**Published:** 2024-11-21

**Authors:** Longxue Chang, Zhichao Liu, Xiaoping Ying, Baxtiyor Kalandarov, Muhammad Ergashev, Xiaohong Tong, Jian Zhang, Jian Jin, Jiezheng Ying

**Affiliations:** 1 State Key Laboratory for Conservation and Utilization of Subtropical Agricultural Resources, College of Life Science and Technology, Guangxi University, Nanning 530004, China; clx17590935230@163.com (L.C.); liuzhichao@ibcas.ac.cn (Z.L.); 2 State Key Laboratory of Rice Biology and Breeding, China National Rice Research Institute, Hangzhou 311401, China; tongxiaohong@caas.cn (X.T.); zhangjian@caas.cn (J.Z.); 3 Agro-Tech Extension and Service Station of Jiangbei District, Ningbo 315033, China; 13566311699@163.com; 4 Rice Research Institute of Uzbekistan, Tashkent 999033, Uzbekistan; baxtiyor1451@gmail.com (B.K.); muhammadsan78@gmail.com (M.E.)

**Keywords:** rice, genes, QTL, lipid metabolism, functional mechanism

## Abstract

Lipids are the basic biological molecules in plants, serving as glycerolipids for cell membranes and triacylglycerols as an energy source. Fatty acids are the major components of plant lipids. Both lipids and fatty acids significantly influence rice quality. Recent studies, through genetic analysis, have made significant progress in uncovering the functional mechanisms and regulatory pathways of lipid metabolism including the biological synthesis and degradation of fatty acids, glycerolipids, and triacylglycerols in rice. Meanwhile, quantitative trait loci (QTLs) identified by analyzing the natural variations of the composition and contents of lipids and fatty acids have been integrated and represented on 12 chromosomes. Lipids play multifaceted roles in the growth and development and stress response of rice. Through metabolic engineering and gene-editing technologies, significant advancements have been made in improving the lipid content in rice grains. These studies highlight the understanding the of molecular basis of lipid metabolism and lay a substantial basis for the genetic improvement of rice quality.

## 1. Introduction

Rice (*Oryza sativa* L.) is one of the major crops in China, with approximately two-thirds of the population relying on it as a staple food [1], and its nutritional value and quality are crucial for human health and well-being. It not only plays a key role in food security and nutrition supply, but also occupies an important position in the agricultural economy of many countries. Rice is a C3 plant with a unique photosynthetic pathway and growth cycle. It can grow under a variety of environmental conditions, including paddy fields and dry land, which makes it widely cultivated around the world. Lipid metabolism plays a key role in plant growth and development and in response to stress, especially in rice, where the composition and content of lipids directly affect the quality and nutritional value of the rice. Moderately adjusting lipid components can enhance the shine, softness, and cooking quality of the rice grains [2]. The fatty acids in rice mainly include oleic acid, linoleic acid, and palmitic acid, which contribute to alleviating conditions such as diabetes, liver diseases, and cardiovascular diseases [3]. Lipids in rice play a crucial role in storage stability; higher levels of unsaturated fatty acids can slow down oxidation during storage, extending shelf life, reducing food loss, and ensuring the safety and nutritional quality of the rice [4]. Additionally, lipids are involved in many critical biological processes in plant growth and development, such as cell division, hormone synthesis, and energy metabolism. Stable lipid content in rice ensures normal plant growth and development. Furthermore, in adverse conditions such as drought, low temperatures, and saline–alkaline soils, higher lipid content can enhance plants’ stress resistance and allow them to better adapt to unfavorable environmental conditions.

In recent years, with the development of molecular biology and genomics technologies, significant progress has been made in the study of rice lipid metabolism. Through genetic analysis and functional genomics research, scientists have identified multiple genes and quantitative trait loci (QTLs) related to lipid metabolism. These studies not only reveal the molecular mechanisms of lipid metabolism but also provide possibilities for improving rice quality through genetic improvement.

This review aims to synthesize the current research progress on the molecular basis of rice lipid metabolism, and explore the role of lipid metabolism in rice and how to optimize lipid composition through genetic improvement to enhance the quality and nutritional value of rice.

### 1.1. Rice Lipid Content, Distribution, and Composition

Rice is rich in nutrients, with its main components being starch, protein, and lipids. Starch is one of the major components, accounting for 85–90% of the total weight of rice. Protein follows, making up 7–12%, while lipids are present in a range of 0.2–3.4%. Additionally, rice contains small amounts of vitamins and trace elements [5]. The lipid content in rice varies depending on factors such as rice variety, growth conditions (temperature at maturity, light intensity, fertilization, etc.), processing methods, and storage conditions [6]. Triglycerides are the primary form of lipid storage in rice, existing in the form of oil bodies within cells. They are mainly distributed in the husk, aleurone layer, embryo, and endosperm. In brown rice, the lipid content ranges from 1.7% to 3.4%, with over 70% found in the embryo, followed by the husk and aleurone layer, and the lowest concentration in the endosperm. In polished rice, the lipid content is 0.1–1.52%, primarily found in the aleurone layer.

Rice lipids can be categorized based on their structural forms into starch lipids (SLs) and non-starch lipids (NSLs). Starch lipids are complexes formed by the interaction of starch and lipids under certain conditions through hydrophobic interactions and hydrogen bonds. They are mainly found in the starchy endosperm, with a content of 0.21–0.76%. The primary free fatty acids that combine with starch are palmitic acid (C16:0) and linoleic acid (C18:2). Non-starch lipids refer to the lipids on the surface of starch granules that can bind with cell membranes and proteins. They are primarily distributed in the aleurone layer, with a content of 2.9–3.4% [7]. The main components of non-starch lipids include free fatty acids and glycerolipids. Free fatty acids are categorized into saturated fatty acids, monounsaturated fatty acids, and polyunsaturated fatty acids. Key fatty acids include oleic acid (C18:1), linoleic acid (C18:2), and palmitic acid (C16:0), along with smaller amounts of stearic acid (C18:0), alpha-linolenic acid (C18:3), myristic acid (C14:0), arachidic acid (C20:0), and trace amounts of lauric acid (C12:0), palmitoleic acid (C16:1), behenic acid (C22:0), and arachidonic acid (C20:4) [8].

### 1.2. Functional Characteristics of Rice Lipids

#### 1.2.1. Roles of Lipids in Rice Growth and Development

During the growth and development of rice, lipid synthesis and metabolism are crucial for its development. Lipids are important components of biological membranes, and lipid metabolism processes, including those of membrane lipids and fatty acids, have significant physiological effects on plant growth, cold resistance, and hormonal regulation [6]. Starch, protein, and lipids are the primary storage substances in rice seeds, and the synthesis and metabolism of lipids have a major impact on seed vitality. Studies have found a significant correlation between the fatty acid composition in rice seeds and seed vitality indicators. For example, the contents of myristic acid, stearic acid, and oleic acid are positively correlated with various vitality indicators, while the contents of palmitic acid, linoleic acid, and alpha-linolenic acid are negatively correlated [9]. The gene *OsLTPL36*, which encodes a lipid transport protein in rice, is mainly expressed in the developing husk and aleurone cells of the endosperm. Downregulation of this gene significantly reduces the seed setting rate and thousand-grain weight, as well as decreases lipids content in grains, thereby inhibiting seed growth vitality [10]. Jasmonic acid (JA), a derivative of fatty acids, is considered an essential plant hormone for growth and environmental adaptation. Studies show that jasmonic acid plays a crucial role in pollen development and fertility, and its synthesis and regulation are closely related to male sterility in plants [11].

#### 1.2.2. Stress Response of Lipids in Rice

The degree of saturation of fatty acids in membrane lipids is closely related to cell cold resistance. Research indicates that membrane lipids and fatty acid metabolism may be important factors in rice cold tolerance. Higher levels of unsaturated fatty acids in membrane lipids lower the phase transition temperature of rice, enhancing its cold resistance [12]. The acyl carrier protein OsMTACP2 enhances rice cold tolerance by engaging in lipid metabolism, particularly in wax synthesis, which facilitates anther and pollen development at low temperatures. *OsMTACP2* is mainly expressed in the tapetum layer and pollen grains of rice anthers, and its loss-of-function mutants are impaired in the formation of anther cuticle and pollen wall development under low temperatures [13]. β-ketoacyl-ACP synthase (KASI), a crucial enzyme in lipid synthesis metabolism, and *OsKASI-2*, is primarily expressed in rice leaves and anthers. The loss of its function results in decreased KAS enzyme activity and unsaturated fatty acid levels, which impairs the unsaturation degree of membrane lipids and increases the sensitivity of rice to chilling stress [14].

In addition to being related to plant cold tolerance, lipid metabolism also affects other stress factors. The rice *HTS1* (high temperature sensitive 1) gene, which encodes a functional β-ketoacyl carrier protein reductase involved in de novo fatty acid biosynthesis, confers heat tolerance to rice by regulating fatty acid biosynthesis and stress signal transduction. The loss of *HTS1* function leads to direct impairment of fatty acid biosynthesis, and the reduction of fatty acid content destroys the integrity and stability of the cell membrane system under heat stress [15]. The *STH1* gene, key to rice salt tolerance, encodes a hydrolase with an α/β-fold domain structure, and research has confirmed that *STH1* functions as a fatty acid hydrolase involved in plant fatty acid metabolism, and affects the integrity and fluidity of the plasma membrane components under salt stress [16]. Phospholipids, vital components of biological membranes, are synthesized via the enzyme CDP-DAG synthase, encoded by the *OsCDS5* gene in rice. A loss of function in *OsCDS5* alters lipid metabolism, leading to enhanced responses to abiotic and biotic stress. The *Oscds5* mutant shows enhanced resistance to rice blast, bacterial blight, and bacterial leaf streak. Mutation of *OsCDS5* promotes the production of reactive oxygen species and increases the expression level of multiple defence-related genes [17].

#### 1.2.3. Roles of Lipids in Rice Quality

Lipids, as significant components of rice, have a notable impact on the cooking quality of rice. In rice, lipids form single-helix complexes with amylose, altering the molecular structure and physicochemical properties of starch. This affects starch characteristics such as gelatinization [18], retrogradation [19], swelling, and adhesiveness [20], which in turn influences rice palatability. The hydrophobic helical cavities in amylose structures can bind with other polar molecules through hydrophobic interactions. When fatty acids form complexes with starch, they reduce the permeability of water molecules to starch granules, thereby inhibiting the swelling and dissolution of starch granules in cold water. Phospholipids, an important class of lipids, affect the swelling and adhesiveness of rice. Research has shown that by regulating the gene *OsPLDα1*, which encodes phospholipase D in rice, the accumulation of lysolipids can be increased, thus improving the cooking quality of rice [21].

On the other hand, the hydrolytic oxidation of fatty acids is a major cause of rice deterioration and rancidity during storage. Studies have indicated that while the total lipid content remains unchanged during rice oxidation, the relative content of various fatty acid components changes [22]. During harvesting, transportation, processing, and storage, physical damage caused by human or mechanical factors can lead to oxidation and decomposition reactions involving fatty acids and lipases, resulting in the formation of unsaturated fatty acids such as oleic acid, linoleic acid, alpha-linolenic acid, and arachidonic acid. These reactions produce corresponding hydroperoxides and release volatile toxic substances like aldehydes and ketones, leading to sourness and odor in rice, which severely affects its edibility and nutritional value [23,24]. Research has found that rice irrigated with molecular hydrogen (hydrogen nanobubble water, HNW) exhibits enhanced antioxidant capacity, reduced lipoxygenase activity, and lower transcript levels, which in turn decreases lipid peroxidation. This results in a noticeable reduction in volatile substances and off-flavors during storage, further improving the antioxidant capacity and storage quality of rice [25].

## 2. Biosynthesis and Metabolism in Rice Lipids

### 2.1. Lipid Biosynthesis

**Figure 1:**
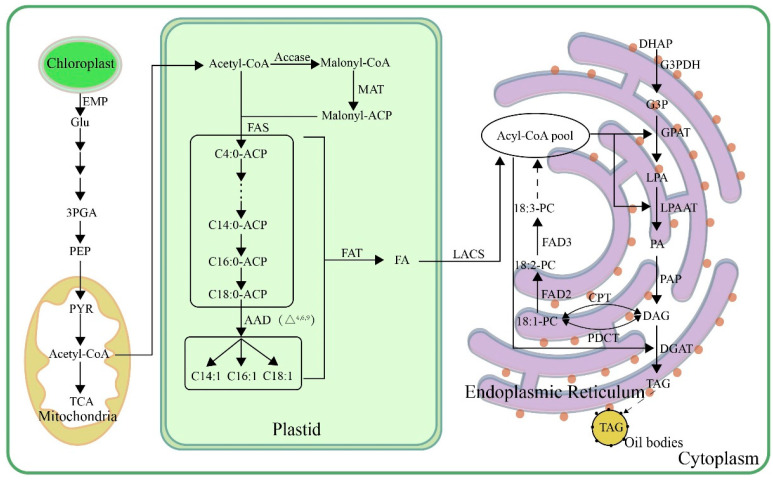
Biosynthetic pathways of TAG in plant seeds. Glu, glucose; 3GPA, 3-phosphoglycerate; PEP, phosphoenolpyruvate; PYR, pyruvic acid; acetyl-CoA, acetyl-coenzyme A; ACCase, acetyl-CoA carboxylase; malonyl-CoA, malonyl-coenzyme A; malonyl-ACP, malonyl-acyl carrier protein; MAT, malonate acyltransferase; FAS, fatty acid synthase; AAD, acyl-ACP desaturase; FAT, acyl-ACP thioesterase; LACS, long-chain acyl-CoA synthetase; DHAP, dihydroxyacetone phosphate; G3PDH, glyceraldehyde-3-phosphate dehydrogenase; G3P, glycerin 3-phosphate; GPAT, glycerol-3-phosphate acyltransferase; LPA, lysophosphatidic acid; LPAAT, lysophosphatidic acid acyltransferase; PA, phosphatidic acid; PAP, phosphatidic acid phosphatase; DAG, diacylglycerol; DGAT, diacylglycerol acyltransferase; TAG, triacylglycerol; PDCT, phosphatidylcholine transferase; CPT, CDP-choline transferase; PC, phosphatidylcholine; FAD2, oleic acid desaturase; FAD3, linoleic acid desaturase.

In plants, glucose synthesized through photosynthesis in leaves is transported to the seeds via the phloem. This glucose is then utilized in various metabolic pathways, including the Embden–Meyerhof pathway (EMP), the pentose phosphate pathway (PPP), gluconeogenesis (GNG), and the tricarboxylic acid cycle (TCA cycle), to provide the raw materials and energy for lipid biosynthesis. The synthesis of plant lipids involves three main processes: fatty acid synthesis, which primarily occurs in plastids, precedes the process of triacylglycerol (TAG) synthesis, wherein free fatty acids are transported to the endoplasmic reticulum for the formation of TAG. Subsequently, oil body formation takes place, during which TAG is encapsulated to form oil bodies, which are then released from the endoplasmic reticulum [26] (Figure 1).

Acetyl-CoA is a precursor for fatty acid synthesis and serves as the donor of all carbon atoms in the fatty acid carbon chain. After being produced through glycolysis, acetyl-CoA is transported to plastids, where it is converted to malonyl-CoA by acetyl-CoA carboxylase. Malonyl-CoA is then transformed into malonyl-ACP by the malonyl CoA-acyl carrier protein transacylase. This malonyl-ACP then condenses with acetyl-ACP in the presence of fatty acid synthase, undergoing a series of condensation, reduction, dehydration, and re-reduction reactions to add two carbon atoms and form butyryl-ACP. Butyryl-ACP undergoes this cycle repeatedly, with each cycle adding two carbon atoms, eventually producing fatty acid-ACP (FA-ACP) with varying chain lengths. Fatty acid synthase is a multi-enzyme complex consisting of β-ketoacyl-ACP synthase (KAS), β-ketoacyl-ACP reductase (KAR), β-hydroxyacyl-ACP dehydratase (HAD), and enoyl-ACP reductase (ER), which sequentially catalyze the condensation, reduction, dehydration, and re-reduction reactions of each cycle. Saturated FA-ACP can be further desaturated into unsaturated FA-ACP by acyl-ACP desaturase. These saturated and unsaturated FA-ACPs are then released from the acyl carrier protein through the action of acyl-ACP thioesterases, resulting in the formation of free fatty acids [27].

Free fatty acids are released on the plastid inner membrane and are then catalyzed by acyl-CoA synthetase on the outer membrane to form acyl-CoA, which is exported from the plastid and enters the endoplasmic reticulum, forming an acyl-CoA pool. Acyl-CoA reacts with glycerol 3-phosphate under the catalysis of glycerol-3-phosphate acyltransferase to form lysophosphatidic acid. LPA can be further acylated by lysophosphatidic acid acyltransferase to produce phosphatidic acid, which is then hydrolyzed by phosphatidic acid phosphatase to form diacylglycerol (DAG) [28]. DAG is a key precursor for the formation of TAG. Beyond the Kennedy pathway, DAG can be interconverted with phosphatidylcholine under the action of cholinephosphotransferase. Additionally, phosphatidylcholine transferase can catalyze the transfer of the phosphatidylcholine group from PC to DAG, resulting in new polyunsaturated DAG [29]. DAG is then converted to TAG by diacylglycerol acyltransferase (DGAT). TAG, formed in the endoplasmic reticulum, cannot remain stable within plant seeds and will associate with oil body proteins to form oil bodies [30].

### 2.2. Lipid Metabolism

In most plants, fatty acids are stored in seeds in the form of triacylglycerols. During seed germination and seedling development, plants rely on the breakdown of stored materials within the seeds to provide energy and substances that promote growth. The biological metabolism of plant lipids includes the hydrolysis of lipids and the oxidation of fatty acids.

**Figure 2:**
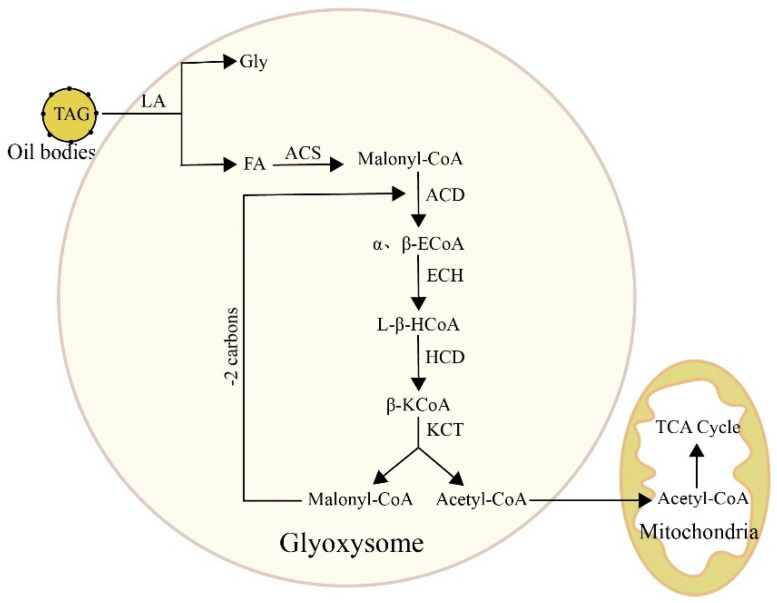
Biological metabolic pathways of TAG in plant seeds. LA, lipase; Gly, glycerol; LOX, lipoxygenase; FFA, free fatty acid; ACS, acyl-CoA synthetase; ACD, acyl-CoA dehydrogenase; α,β-ECoA, α,β-enoyl-CoA; ECH, β-enoyl-CoA hydratase; L-β-HoA, L-β-hydroxyacyl-CoA; HCD, L-β-hydroxyacyl-CoA dehydrogenase; β-KCoA, β-ketoacyl-CoA; KCT, β-ketoacyl-CoA thiolase.

Lipase is a key enzyme in the lipid hydrolysis process, breaking down lipids into glycerol and fatty acids. The free fatty acids are oxidized by lipoxygenase within the glyoxysome to form acyl-CoA and acetyl-CoA, undergoing β-oxidation [31]. Key enzymes in the β-oxidation process include acyl-CoA dehydrogenase (ACD), β-enoyl-CoA hydratase (ECH), L-β-hydroxyacyl-CoA dehydrogenase (HCD), and β-ketoacyl-CoA thiolase (KCT). After hydrolysis, fatty acids are activated to acyl-CoA by acyl-CoA synthetase (ACS), then transported into the mitochondria by carnitine, and undergo ACD dehydrogenation, ECH hydration, HCD dehydrogenation, and KCT thiolysis to produce acyl-CoA and acetyl-CoA. Acyl-CoA can continue to cycle in β-oxidation, while acetyl-CoA enters the glyoxysome to produce succinate, aldehyde acid, and malate, with succinate being used for sugar synthesis [32]. Aldehyde acid can further oxidize to form volatile aldehydes and ketones (Figure 2). Additionally, under aerobic conditions, unsaturated fatty acids also undergo oxidation to produce hydrogen peroxide, which is further oxidized to aldehydes, ketones, and acids [33]. The combined action of LA and LOX decreases the storage quality of rice.

## 3. Genetic Basis for Controlling Rice Lipid and Fatty Acid Content

### 3.1. Identification of Genes/QTLs Controlling Rice Lipid and Fatty Acid Content

Quantitative trait loci (QTLs) refer to certain regions in the genome that contain one or more genes associated with phenotypic variation in quantitative traits. QTL analysis is a statistical method that links two types of information, phenotypic data and genotypic data (molecular markers), to explain the genetic basis of variation in complex traits. Rice lipid and fatty acid content are typical quantitative traits, with extensive genetic variation observed among different rice species, subspecies, and varieties [34,35,36]. By analyzing the natural variation in oil and fatty acid content among different rice materials, the key genes/QTLs controlling lipid metabolism can be identified, and their mechanisms and regulatory pathways can be elucidated. This provides a genetic basis for improving rice growth and development and enhancing rice quality. Multiple quantitative trait loci (QTLs) related to rice lipid content have been reported in various rice populations. Wu et al. [37] used an RIL population from the cross between Asominori and IR24 and located a QTL for rice oil content in the R1629-XNpb37 interval on chromosome 10, explaining 19% of the genetic variation. Hu et al. [38] constructed a DH population from the parents Gui630 and 02428 and identified three QTLs for rice oil content on chromosomes 1, 2, and 5, with *qRFC-2* and *qRFC-5* contributing 24% and 26%, respectively. Using the RIL population derived from the cross between Xieqingzao B and Miyang 46 to detect rice lipid content, four QTLs associated with rice lipid content were mapped on chromosomes 3, 5, 6, and 8. Among them, *qLc-5* exhibited the highest contribution rate of 12.0% [39]. An RIL population derived from the Japonica variety Asominori and the Indica variety IR24 was employed to examine lipid content in rice grains during the filling stage. Ten conditional QTLs controlling lipid content were identified, with four detected in the early filling stage, two in the middle stage, one in the late stage, and three at maturity. All conditional QTLs were detected only at specific stages, indicating that the synthesis of grain oil is jointly controlled by genes expressed at different stages [40]. Liu et al. [41] conducted QTL analysis for rice oil content using a DH population derived from the cross between the parents Zhenshan 97B and Wuyujing 2, as well as two backcross populations. They identified ten QTLs controlling oil content on chromosomes 1, 3, 5, and 9, with *qCFC5* being the major QTL located in the RM87-RM334 interval on chromosome 5, explaining 10.67% to 25.77% of the phenotypic variation and detected in all three populations. The RIL population constructed by Sasanishiki/Habataki detected three major QTLs controlling lipid content on chromosomes 3, 6 and 11, explaining that the range of phenotypic variation rate was 16.33–21.03% [42]. The RIL population constructed by Minghui 63 and the high-quality Thai fragrant rice KDML105 mapped three QTLs controlling crude lipid content on chromosomes 1, 7 and 8, of which *qFC-7* had the highest contribution rate of 7.30% [43]. Ying et al. [44] used F_2_ and F_2:3_ populations from the cross between FAZ2 and JZ1560, analyzed fatty acid content using gas chromatography, and identified 29 QTLs controlling rice lipid and fatty acid content, explaining 5.28% to 37.93% of the phenotypic variation. They also inferred candidate genes for 11 of these QTLs based on information from Arabidopsis lipid metabolism genes. Kim et al. [45] performed QTL analysis for lipid content using an F3 population from the cross between Samgang and the high-oil mutant “P31-2-2-2-B-B,” identifying a major QTL *qRLC5* on chromosome 5, explaining 20.00% of the phenotypic variation, and indicating that lipid content is a quantitative trait controlled by multiple genes. QTLs related to rice quality were analyzed using a DH population from the cross between Cheongcheong and Nagdong. Three QTLs for lipid content were located on chromosomes 2, 3, and 6, namely qLip-2, qLip-3, and qLip-6, with each contributing 30.00% [46]. Data from 35 mapping populations were collected and compiled, and a meta-analysis approach was used to obtain “true” QTLs, establishing a consistent map for QTLs related to rice lipid content. Twelve lipid content related QTLs were identified on chromosomes 3, 5, 6, 7, and 8, providing a theoretical basis for the discovery and cloning of rice lipid content-related genes [47]. Zhou et al. [48] conducted a GWAS for oil-related traits in 305 Indica and 178 Japonica rice subpopulations, identifying 46 QTLs. They also constructed RIL populations from ZS97/MH63, B805D/H6S, and XH/HD9802S and identified 53 QTLs associated with oil traits with LOD > 3, including 16 QTLs that co-localized with significant loci identified in the GWAS analysis. The NIL population constructed by 88B-2/Hua 2613S mapped four QTLs on chromosomes 1, 5, 6 and 7, of which *qFC6* was the main QTL, explaining 55% of the phenotypic variation [49]. Mai et al. [50] performed a GWAS analysis on the components and lipid content of 161 Vietnamese rice varieties, evaluating QTLs related to C18:1 and C18:2 unsaturated fatty acid content using LD analysis. They detected seven QTLs mainly distributed on chromosomes 1, 2, 7, and 11.

**Figure 3:**
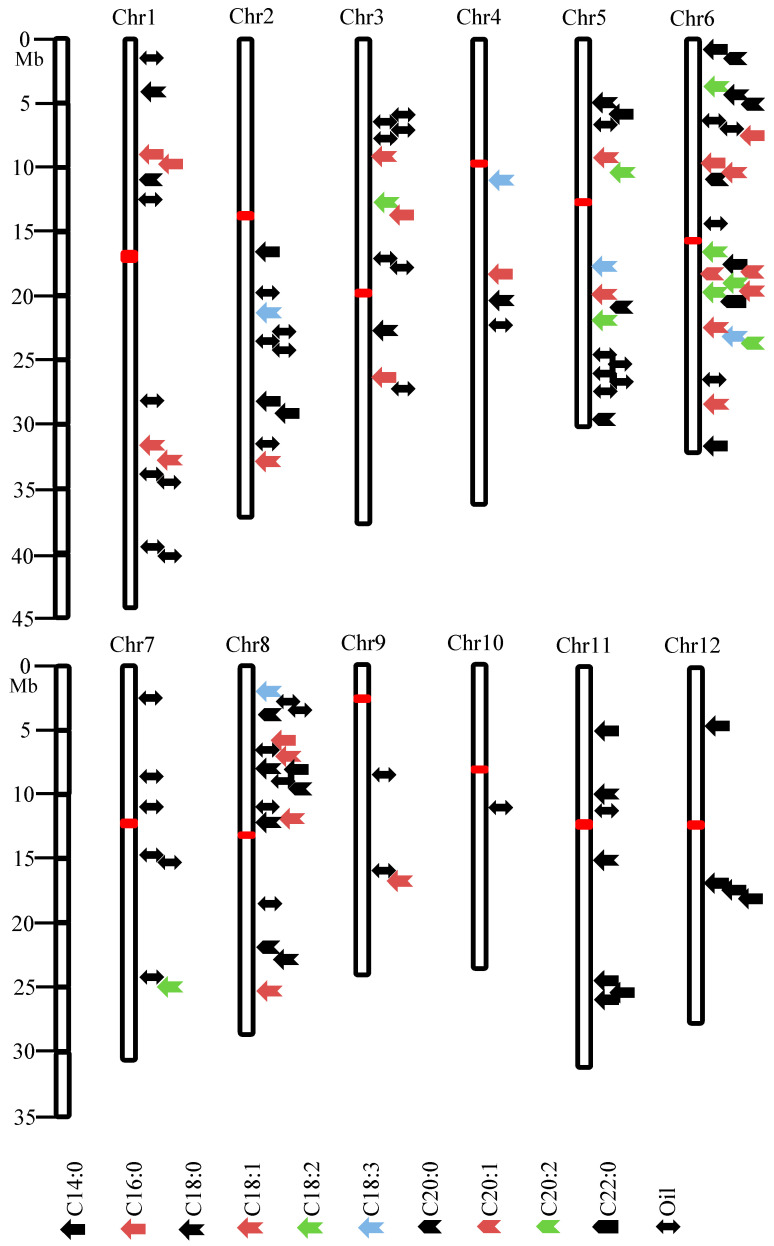
Distribution of QTLs controlling lipid and fatty acid content on 12 chromosomes of rice. Red thick line, centromere.

Currently, a substantial number of QTLs related to rice lipid and fatty acid content have been identified, distributed across all 12 rice chromosomes (Figure 3). These QTLs are primarily associated with lipid components and content. For example, Liu et al. [41] identified the QTLs *qCFC1*, *qCFC3*, *qCFC5*, and *qCFC7* in both DH and BCF1 populations, with several of these QTLs being consistently detected across both populations. Huang et al. [43] found that *qFC-1* detected in their RIL population co-localized with *qFC-1* identified by Xia et al. [48] in an NIL population. Additionally, *qLC-6* identified by Yu et al. [49] in an RIL population co-localized with *qCFC6a* detected by Liu et al. [41] in a DH population, while *qLC-8* identified by Yu et al. in their RIL population co-localized with *qFC-8* identified by Huang et al. [43] in their RIL population. Furthermore, QTLs associated with C14:0 content are predominantly found on chromosomes 2, 6, 11, and 12, with detection in F_2_, F_2:3_, and NIL populations. Ying et al. [44] repeatedly detected the QTLs *qmyr2-1*, *qmyr6*, and *qmyr12* in the F2 and F2:3 populations. Similar distribution patterns are observed for QTLs related to C16:0, C18:0, C18:1, C18:2, C18:3, C20:0, C20:1, C20:2, and C22:0 content.

Although these QTLs are widely distributed across all 12 chromosomes, most are still in the preliminary mapping stages with relatively large intervals. Therefore, more precise localization is required to facilitate their application in molecular-assisted breeding.

### 3.2. Molecular Regulation of Rice Lipid Metabolism

The biosynthesis of rice fatty acids is a complex process regulated by multiple genes, with several genes already reported to be involved in fatty acid synthesis and metabolism. The expression and analysis of three ω-3 fatty acid desaturase (FAD) genes in rice were conducted, and it was confirmed that linoleic acid (C18:2) can be converted to α-linolenic acid (C18:3) by OsFAD3. The content of C18:3 in seeds overexpressing OsFAD3 was increased by 23.8–27.9-fold [51]. Zapin et al. [52] performed bioinformatics, expression, and transgenic analyses of four putative fatty acid desaturase (*OsFAD2*) genes in rice, finding that RNAi-mediated suppression of *OsFAD2* expression led to increased oleic acid content in grains, with reduced linoleic and palmitic acid levels, indicating a key role of *OsFAD2* in converting oleic acid (C18:1) to linoleic acid (C18:2). LOX3, an isozyme of LOX, primarily catalyzes the formation of 9-HPOD. It was found that inhibiting the expression of LOX3 in rice endosperm and reducing the content of volatile substances such as hexanal, pentanal, and pentanol in rice could improve rice storage without affecting rice phenotype and yield [53], which has a considerable application prospect in agricultural production. Zhang et al. [54] demonstrated that introducing the C4 photosynthetic gene phosphoenolpyruvate carboxylase (PEPC) into rice promotes fatty acid accumulation. They isolated a mutant *fse1* from rice, which exhibited reduced starch content and altered starch physicochemical properties, with increased lipid and phosphatidic acid levels [55]. This suggests that FSE1 is a phospholipase-like protein controlling the synthesis of galactolipids in rice endosperm, providing a new link between lipid metabolism and starch synthesis during endosperm development. Zhou et al. [48] analyzed fatty acid composition and oil content in 533 rice varieties, identifying 26 candidate genes for rice fatty acid content QTLs, with four genes (*PAL6*, *LIN6*, *MYR2*, and *ARA6*) associated with natural variation in fatty acid composition. It was demonstrated that the distribution of the rice soluble gibberellin receptor GID1 is regulated by phospholipase D (PLD) and phosphatidic acid (PA) [56]. Knockout plants of *pldα6* were shown to have reduced sensitivity to GA compared to the wild type (WT), while PA was found to restore the normal GA response in the mutants. It was found that PLDα6 and PA positively mediate rice GA signaling by binding PA to GID1 and facilitating its translocation into the nucleus. Lipid metabolism, including membrane lipids and fatty acids metabolism, is a crucial factor for cold tolerance in rice [12]. Lipidomics data confirmed the importance of membrane lipid remodeling and fatty acid unsaturation for rice adaptation to cold stress, with upregulation of phosphatidic acid phosphatase (*PAP2*) inhibiting excessive accumulation of phosphatidic acid (PA), leading to increased levels of phosphatidylcholine (PC), phosphatidylethanolamine (PE), and phosphatidylglycerol (PG), thereby preventing membrane phase transition under cold stress. They studied the key transcription factor WRI1, which regulates seed oil biosynthesis in *Arabidopsis*, and found that overexpressing *OsWRI1* in rice increased oil content in rice endosperm and leaves, altered fatty acid composition, and elevated the expression levels of genes related to glycolysis and fatty acid synthesis. Ectopic expression of *OsWRI1a* in rice promoted oil biosynthesis but also resulted in abnormal plant growth and development [57]. Xia et al. [49] identified a QTL, *qFC6*, influencing fat content through association and linkage analysis, with the candidate gene *Wx* negatively regulating fat content in rice, aiding in the understanding of the genetic basis of rice fat content and improving rice eating quality. Mai et al. [50] performed a GWAS analysis on oil content in 161 Vietnamese rice varieties, identifying two genes involved in fatty acid synthesis (*OsKASI* and *OsGAPDH*) and three genes affecting fatty acid content (*OsARF*, *OsFAD*, and *OsMADS29*). They also conducted RNA sequencing on two rice lines with similar lipid content and agronomic traits but differing in lipid and flavor profiles (FH and FL) [58], finding significantly higher expression of genes related to fatty acid synthesis and elongation (*Os11g0102500* and *Os11g0210500*) in FH grains, while genes associated with fatty acid degradation (*Os04g0540600*, *Os07g0417200*, and *Os04g0573900*) were downregulated. Liquid chromatography analysis revealed higher relative contents of palmitic acid and oleic acid in FH grains. Zhang et al. [14] found that β-ketoacyl-ACP synthase (KASI) plays a crucial role in regulating membrane lipid unsaturation and maintaining membrane structural homeostasis under low-temperature stress, with disruption of *OsKASI-2* leading to reduced KAS enzyme activity and unsaturated fatty acid levels. In rice, controlling the expression of the oil synthesis rate-limiting enzyme gene *AtDGAT1* with an endosperm-specific promoter, while knocking out the key starch synthesis gene *OsAGPL2* and key gene *OsMTSSB1* controlling the thickness of the aleurone layer shifted carbon flow from starch to oil, enhancing oil biosynthesis and increasing oil content in rice by over fivefold [59].

## 4. Summary

Lipids, as the third major nutritional component in rice, significantly impact rice quality and are of considerable interest in rice quality improvement and breeding. With ongoing research into rice lipids, their nutritional value and functional characteristics are becoming increasingly understood. The content of rice lipids is correlated with amylose content, and the interaction between lipids and starch can influence the functional properties of starch, thereby affecting the cooking and eating quality of rice [18,19,20]. While the digestion performance of starch in the human body is well understood, the impact of lipids on rice digestion is rarely reported [60].

The fatty acid content in rice is closely related to seed vitality. The breakdown of lipids in seeds produces glycerol and free fatty acids. During storage, an increase in temperature raises the fatty acid content in seeds, leading to lipid peroxidation and reduced germination rates. Therefore, rice breeding should focus on selecting germplasms with lower fatty acid values. The composition of free fatty acids in rice is complex, with recent reports mainly focusing on crude fat content or related functions. There are fewer studies on individual free fatty acid components, primarily due to its extremely low content in rice, making it difficult to observe related phenotypes. Existing methods for detecting rice lipid content have several limitations, such as high cost, complex procedures, and significant result variability. Thus, establishing rapid and accurate detection methods remains a critical issue for current research.

The synthesis and metabolism of rice lipid is a complex biological process regulated by multiple genes. Despite progress in understanding the regulatory mechanisms of lipid synthesis through genetics, molecular biology, biochemistry, and multi-omics analyses, there is still limited deeper understanding of this process, particularly regarding lipid metabolism. Current research is mostly focused on the fatty acid oxidizing enzymes LOX and their isoforms, with some QTLs identified for fatty acid synthesis, but these are primarily at preliminary stages of mapping and require fine mapping for application in molecular breeding.

Lipid metabolism is crucial for rice to cope with environmental stresses. Specific lipid components and metabolic pathways play a role in low temperatures, drought, salt stress, and resistance to diseases and pests. Understanding the role of lipid metabolism in adversity helps breeders identify and utilize genes related to lipid metabolism to develop rice varieties that are more resistant to stress and increase the yield of rice seed production. Lipid metabolism is also involved in the reproductive development of rice. Lipid layers, including the anther cuticle and pollen exine, are essential for the development of male reproductive organs. Deficiencies in lipid synthesis can result in pollen infertility, potentially leading to plant sterility. While numerous genes associated with lipid metabolism have been identified and their roles in male sterility in rice and other plants have been elucidated, it is possible that these studies do not encompass all relevant biological processes [61]. Furthermore, enhancing lipid content in rice grains through multi-gene engineering might impact other physiological traits of rice. The challenge of overcoming the oxidative susceptibility and instability of high-oil-content rice lipids remains to be addressed.

To advance the efficient research and regulation of rice lipid synthesis and genetics, future studies can employ various strategies. Utilizing advanced technologies such as genomics, transcriptomics, metabolomics, and proteomics will provide deeper insights into the regulatory networks of rice lipid synthesis, identify key regulatory genes, and elucidate their mechanisms, thus offering a theoretical and technical foundation for precise breeding. Additionally, combining gene editing and overexpression technologies to validate and functionally analyze key genes in the lipid synthesis regulatory network will reveal their regulatory mechanisms. Establishing comprehensive systems biology models to fully elucidate the regulatory networks and key nodes will effectively integrate molecular breeding with traditional breeding methods, facilitating the improvement of rice germplasm quality and enhancing breeding efficiency.
